# Adrenocortical Carcinoma Discovered with Point-of-care Ultrasound

**DOI:** 10.5811/cpcem.2021.5.51875

**Published:** 2021-10-05

**Authors:** Mark McIntyre, Michael I. Prats

**Affiliations:** Ohio State University College of Medicine, Department of Emergency Medicine, Columbus, Ohio

**Keywords:** ultrasound, imaging, malignancy, oncology, adrenocortical carcinoma

## Abstract

**Case Presentation:**

A 34-year-old woman presented to the emergency department with bilateral lower extremity edema and shortness of breath. She had been seen by her primary care provider. Lab work and a follow-up with endocrinology had been unrevealing. Using point-of-care ultrasound we identified a cystic mass in the right upper quadrant prompting further imaging.

**Discussion:**

Abdominal and pelvic computed tomography confirmed a mass in the right posterior liver, which was later identified as an adrenocortical carcinoma. Ultrasound is an important diagnostic tool in the setting of lower extremity edema and can be used to assess for heart failure, liver failure, obstructive nephropathy, venous thrombosis, and soft tissue infection. In this case, ultrasound helped expedite the diagnosis and treatment of a rare malignancy.

## CASE PRESENTATION

**Image 1:**
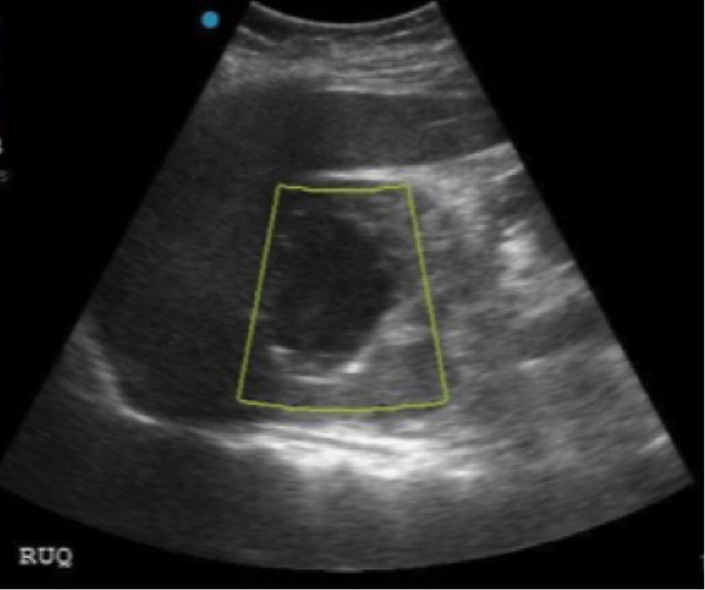
Upper quadrant mass seen on emergency physician-performed point-of-care ultrasound. Color Doppler demonstrating no flow (yellow box).

**Image 2:**
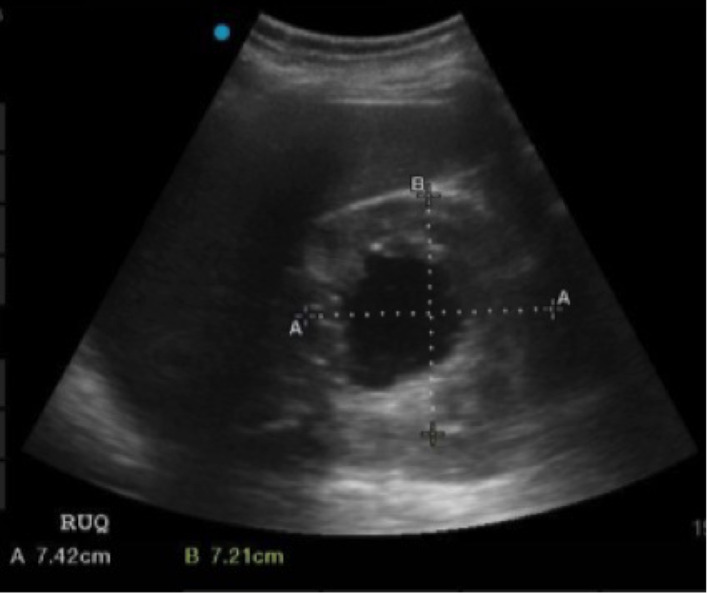
Right upper quadrant mass seen on emergency physician-performed point-of-care ultrasound. Measurements showing 7.42 × 7.21 cm in cephalad-caudal and lateral dimensions, respectively.

**Image 3:**
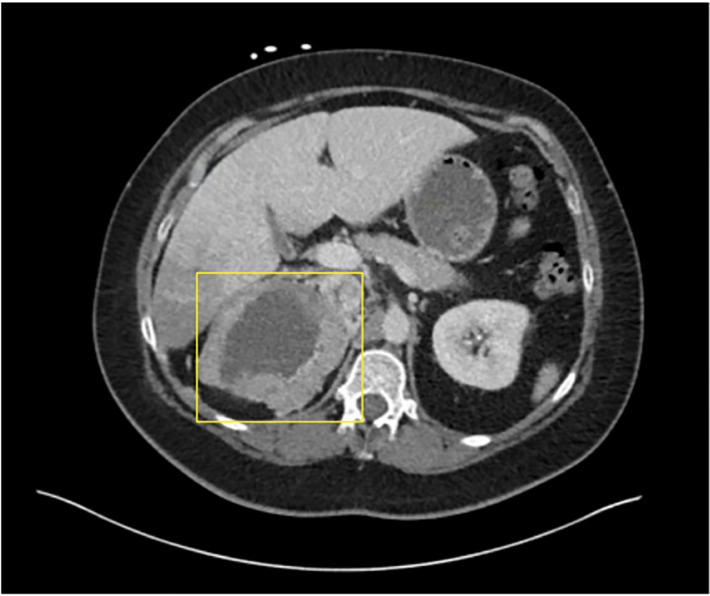
Axial computed tomography demonstrating cystic mass in right upper quadrant (yellow box).

A 34-year-old woman presented to the emergency department (ED) with progressive leg swelling. Thorough laboratory testing as an outpatient and in the ED was unrevealing. To evaluate further the causes of her dyspnea and edema, we performed a point-of-care ultrasound (POCUS) of the heart, lungs, and abdomen. While evaluating for evidence of ascites, a large mass with anechoic center was identified in the right upper quadrant (Video, Images 1 and 2). This prompted computed tomography (CT) of the abdomen and pelvis, which confirmed a 7.9 × 9.1 × 8.7 centimeter mass arising from the right posterior liver, extending into the inferior vena cava with an associated near-occlusive tumor thrombus (Image 3). During admission, a biopsy was performed revealing adrenocortical carcinoma (ACC).

## DISCUSSION

Adrenocortical carcinomas can present as either non-secreting or hormone secreting.^1^ The diagnostic workup for ACC includes the measurement of steroid hormones produced by tumor, imaging via contrast-enhanced CT or magnetic resonance, and biopsy if indicated.^2^ An ACC can be quite large, which incurs a higher risk of complications such as vasculature obstruction, related to the size of the malignancy.^3^ Thus, it is important to identify these tumors expeditiously and begin treatment as soon as possible. The sonographic appearance of ACC is a large, heterogeneous solid or cystic mass positioned adjacent to the kidney.^4^

**CPC-EM Capsule**

What do we already know about this clinical entity?

*Adrenocortical carcinomas present with a variety of symptoms. Typical diagnostic workup includes measuring steroid hormones, computed tomography or magnetic resonance imaging, and biopsy.*

What is the major impact of the image(s)?

*In these images, the utility of point-of-care ultrasound (POCUS) as a diagnostic tool is demonstrated in the setting of unclear symptom etiology.*

How might this improve emergency medicine practice?

*Using POCUS can help narrow differential diagnosis, identify symptom etiology, and expedite treatment.*

There is a broad differential diagnosis for a patient presenting to the ED with lower extremity edema. Point-of-care ultrasound has proven useful in diagnosing many of these etiologies such as acute heart failure, ascites, obstructive nephropathy, venous thromboembolic disease, and soft tissue infection.^5^ In this case, a systematic POCUS evaluation revealed an unexpected cause for the patient’s symptoms, thus expediting her workup and treatment. Multiorgan POCUS is a reasonable early step in the diagnostic management of undifferentiated lower extremity edema. Emergency physicians should be able to recognize concerning sonographic findings and pursue the next steps in diagnostic testing for abdominal neoplasms.

## Supplementary Information

Video Right upper quadrant mass. Ultrasound transducer held in the posterior axillary line demonstrating a circular mass with an anechoic center (arrow) as the beam fans from anterior to posterior.
